# Global Analysis of Gene Expression Profiles in Developing Physic Nut (*Jatropha curcas* L.) Seeds

**DOI:** 10.1371/journal.pone.0036522

**Published:** 2012-05-04

**Authors:** Huawu Jiang, Pingzhi Wu, Sheng Zhang, Chi Song, Yaping Chen, Meiru Li, Yongxia Jia, Xiaohua Fang, Fan Chen, Guojiang Wu

**Affiliations:** 1 Key Laboratory of Plant Resources Conservation and Sustainable Utilization, South China Botanical Garden, Chinese Academy of Sciences, Guangzhou, People’s Republic of China; 2 Graduate University of the Chinese Academy of Sciences, Beijing, People’s Republic of China; 3 BGI-Shenzhen, Shenzhen, People’s Republic of China; 4 National Center for Plant Gene Research, Key Laboratory of Molecular and Developmental Biology, Institute of Genetics and Developmental Biology, Chinese Academy of Sciences, Beijing, People’s Republic of China; Lawrence Berkeley National Laboratory, United States of America

## Abstract

**Background:**

Physic nut (*Jatropha curcas* L.) is an oilseed plant species with high potential utility as a biofuel. Furthermore, following recent sequencing of its genome and the availability of expressed sequence tag (EST) libraries, it is a valuable model plant for studying carbon assimilation in endosperms of oilseed plants. There have been several transcriptomic analyses of developing physic nut seeds using ESTs, but they have provided limited information on the accumulation of stored resources in the seeds.

**Methodology/Principal Findings:**

We applied next-generation Illumina sequencing technology to analyze global gene expression profiles of developing physic nut seeds 14, 19, 25, 29, 35, 41, and 45 days after pollination (DAP). The acquired profiles reveal the key genes, and their expression timeframes, involved in major metabolic processes including: carbon flow, starch metabolism, and synthesis of storage lipids and proteins in the developing seeds. The main period of storage reserves synthesis in the seeds appears to be 29–41 DAP, and the fatty acid composition of the developing seeds is consistent with relative expression levels of different isoforms of acyl-ACP thioesterase and fatty acid desaturase genes. Several transcription factor genes whose expression coincides with storage reserve deposition correspond to those known to regulate the process in Arabidopsis.

**Conclusions/Significance:**

The results will facilitate searches for genes that influence de novo lipid synthesis, accumulation and their regulatory networks in developing physic nut seeds, and other oil seeds. Thus, they will be helpful in attempts to modify these plants for efficient biofuel production.

## Introduction

Physic nut (*Jatropha curcas* L.) is a drought-resistant, non-food oilseed plant that meets many of the requirements for commercial biodiesel production [1]–[4]. Further, following the recent sequencing of its genome and the development of expressed sequence tag (EST) libraries by ourselves and other research groups [5]–[6] it is now a valuable model plant for studying carbon assimilation in the endosperms of oilseeds such as castor bean.

In mature physic nut seeds, triacylglycerols (TAGs) are the major storage compounds [4]. In oilseed plants such as *Brassica napus* L. and Arabidopsis, starch is synthesized at an early stage, while TAGs and storage proteins are mainly synthesized during the seed-filling phase of seed development [7]–[8]. The biosynthesis and accumulation of TAGs from sugars involves hundreds of enzymes acting in several metabolic pathways. The common flux model for TAG synthesis in oilseeds includes the absorption of sugars into seed cells, their breakdown through both cytosolic and plastidic glycolytic pathways, exchange of the intermediates between the cytosol and organelles (such as plastids and the endoplasmic reticulum), starch turnover, malonyl–CoA and fatty acid (FA) synthesis, TAG assembly and oil body formation [9]. In the model plant species Arabidopsis, there are over 800 genes encoding lipid metabolism-related proteins [10]–[11].

Several transcription factor genes involved in seed development and reserve substance accumulation have been identified in Arabidopsis. WRINKLED11 (WRI) regulates several genes encoding late glycolysis enzymes and the plastidial FA biosynthetic machinery [8], [12]–[13]. ABSCISIC ACID INSENSITIVE 3 (ABI3), LEAFY COTYLEDON 1 (LEC1), LEC2 and FUSCA 3 (FUS3) interactively form complex networks that regulate multiple aspects of seed development, including storage reserve accumulation [13]–[20]. ABI4 regulates lipid breakdown in Arabidopsis seed germination [21] and the transcription of acyl-CoA:diacylglycerol acyltransferase 1 (which catalyzes formation of triglycerides from diacylglycerol and acyl-CoA) under low-N conditions [22]. ABI5 activates several late embryogenesis-abundant genes during seed maturation [23]–[24].

Transcriptome analysis of developing seeds can provide fundamental molecular understanding of processes such as embryogenesis, seed filling, maturation, and seed quality. Several such analyses of developing physic nut seeds, using expressed sequence tags (ESTs), have provided limited information on storage accumulation in the seeds [25]–[26]. To extend this information we have characterized seed developmental stages in the species. We then exploited the abundant genomic resources available for physic nut to analyze transcriptomic changes in developing seeds using next-generation sequencing-based digital gene expression tag profiling to establish timeframes of key metabolic processes related to reserve accumulation and their regulators.

## Results

### Physiology of Physic Nut Seed Development

**Figure 1:**
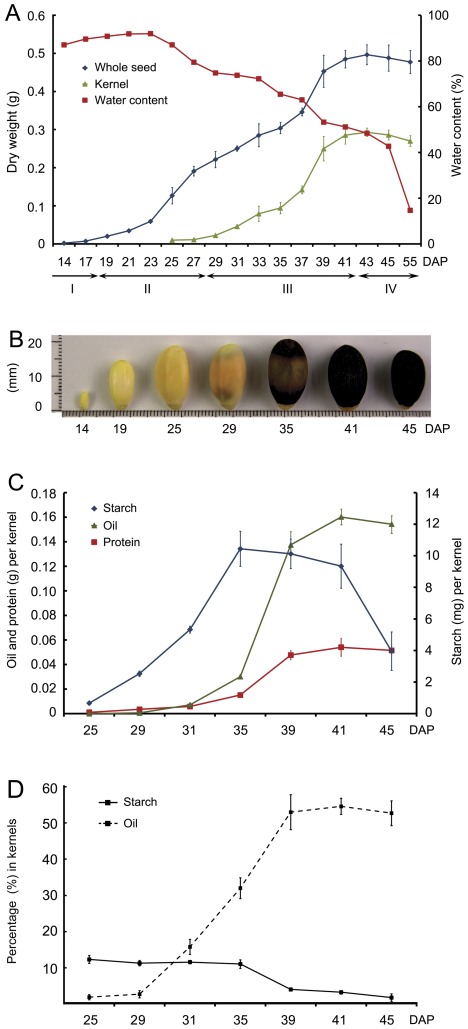
Characterization of physic nut seed development stages. A: Changes in dry weight per seed, dry weight per kernel and water content of whole seeds from 14 to 55 days after pollination (DAP). The seed coat and kernel can be hardly separated before 24 DAP. The four following phases are indicated: I, histodifferentiation; II, seed coat development; III, seed filling; and IV, desiccation. Data are means ±SD of three replicates of 15 seeds. B: Appearance and size of physic nut seeds from 14 to 45 DAP. C: Accumulation kinetics of the storage compounds in kernels during seed development. Values are means (±SD) of three biological replicates. D: Changes in percentage contents of starch and oil in kernels during seed development. Values are means (±SD) of three biological replicates.

To establish a framework for global analysis of gene expression profiles of the developing seeds, we initially defined four developmental stages, from embryogenesis to seed dispersal, characterized by distinct physiological events and associated changes in seed coat color, seed size, whole seed dry weight, kernel dry weight, and moisture status (Figure 1A). The first phase is the histodifferentiation or embryogenesis stage, during the first 17 days after pollination (DAP), under growth conditions at the harvesting site. At this stage the seeds have a water content of ca. 90% (fresh weight), and are milky white or faintly yellow. The second phase, between 17 and 29 DAP, is associated with rapid increases in seed volume and seed coat dry matter, the seeds are yellow to saffron yellow in color, and the seed coat hardens. The third (seed-filling) stage phase, from 29 to 41 DAP, is associated with a rapid increase in kernel dry mass, and a change in the seed coat color to black. Finally, during the late-maturing or desiccation stage there is a sharp declined in seed water content (Figure 1A, 1B). Under our field conditions, starch rapidly accumulated from 25 to 35 DAP, and then declined during the seed-filling stage (Figure 1C). The amount of starch, as a percentage of kernel mass, fell from ca. 11% at 35 DAP to ca. 4% at 39 DAP and ca. 2% at 45 DAP (Figure 1D). The oil and protein accumulation rates were high between 29 and 41 DAP (Figure 1C), in accordance with the rapid increase in kernel dry weight observed during this period (Figure 1A). The percentage contribution of total oils to the kernel mass increased from ca. 3% at 29 DAP to ca. 55% at 41 DAP (Figure 1D).

**Figure 2:**
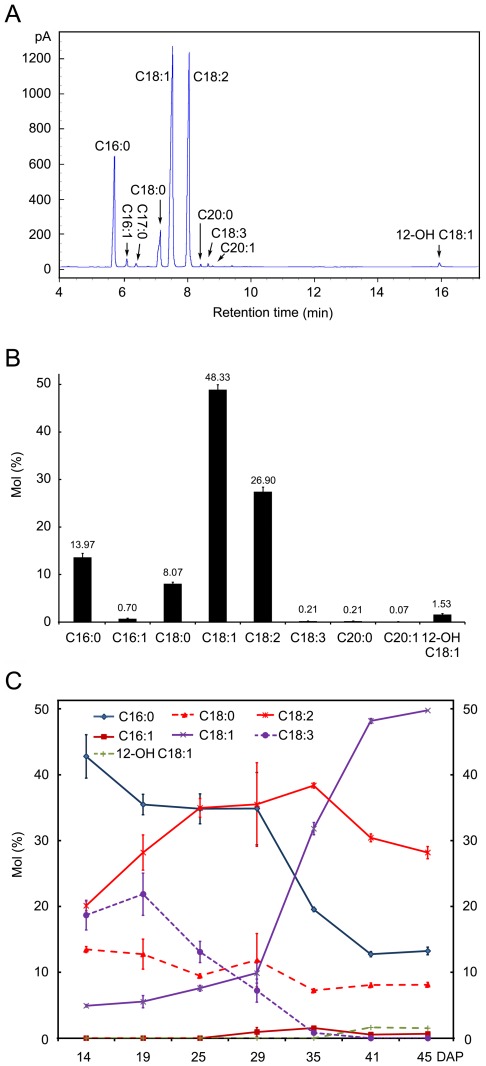
Changes in fatty acid composition during physic nut seed development. A: Gas chromatography of fatty acids in mature physic nut seeds. The peaks were identified by mass spectrometry. B: Fatty acid composition in mature physic nut seeds. Each data point represents the mean (±SD) of three biological replicates. C: Changes in fatty acid composition during seed development. Fatty acids were extracted from whole seeds sampled 14 and 19 days after pollination (DAP) and from kernels of seeds sampled 25 to 45 DAP. Each data point represents the mean (±SD) of three biological replicates. C17∶0 was used as both a seed lipid extraction and transmethylation internal standard. C16∶0, palmitic acid; C16∶1 palmitoleic acid; C18∶0, stearic acid; C18∶1, oleic acid; C18∶2, linoleic acid; C18∶3, linolenic acid; C20∶0, arachidic acid; C20∶1, gondoic acid; 12-OH C18∶1, 12-hydroxyoleic acid (ricinoleic acid).

Our fatty acid analyses confirm previous findings [4] that the most important fatty acids in physic nut oil are palmitic acid (C16∶0), stearic acid (C18∶0), oleic acid (C18∶1) and linoleic acid (C18∶2) (Figure 2A, 2B). Minor amounts (accounting for no more than 3% of the total fatty acids in oils of the mature seeds) of palmitoleic acid (C16∶1), linolenic acid (C18∶3), arachidic acid (C20∶0), gondoic acid (C20∶1), and 12-hydroxyoleic acid (ricinoleic acid, 12-OH C18∶1) were also detected (Figure 2A, 2B). However, the fatty acid composition varied dramatically in different developmental stages of the seeds. Levels of C18∶1 increased, while levels of C16∶0 and C18∶3 decreased as the seeds developed, and C18∶3 contents were very low in mature seeds. C18∶2 levels initially increased as the seeds developed, then declined during the late maturation stage. 12-hydroxyoleic acid appeared during the late seed-filling stage (Figure 2C).

### Strategy for the Global Analysis of Gene Expression Profiles of Developing Seeds

For the genome-wide analysis of gene expression in the developing physic nut seeds, we examined changes in the transcriptome of seeds at seven developmental points using next-generation sequencing-based Digital Gene Expression tag profiling [27]. The seven points were during the following stages: early seed development (histodifferentiation) (S1, 14 DAP), early increase of seed dry-weight (S2, 19 DAP), rapid increase of seed coat dry-weight (S3, 25 DAP), early increase of kernel dry-weight (S4, 29 DAP), rapid increase of kernel dry-weight (S5, 35 DAP), late kernel dry-weight increase (S6, 41 DAP), and desiccation (S7, 45 DAP) (Figure 1A, 1B). ANOVA identified nine clusters of the relative abundance of 11412 detected genes during the seven stages (Table S1). We then examined in detailed changes in the expression of genes pertaining to carbon flow, storage reserve accumulation and their associated transcription factors.

### Sucrose Hydrolysis and Sugars/Sugar-phosphate Translocation-related Genes

**Figure 3:**
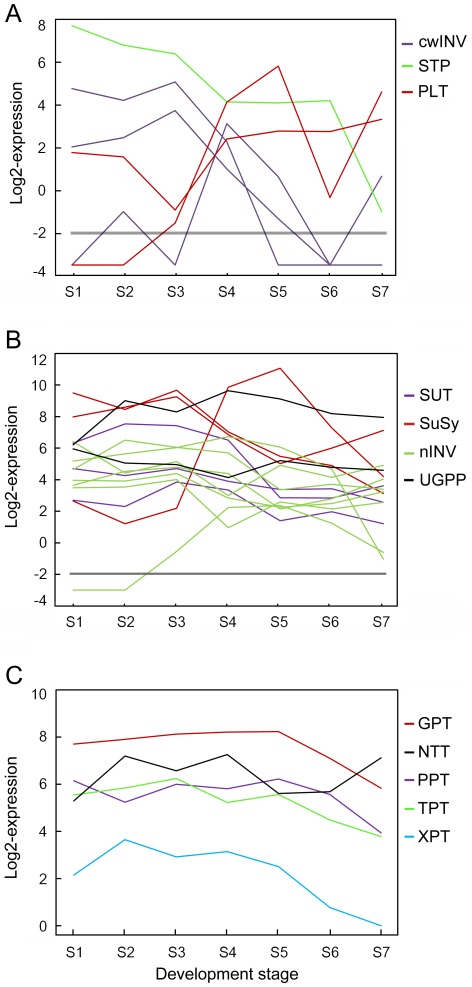
Overview of expression profiles of sucrose absorption and sugar-phosphate transport genes in developing physic nut seeds. A: Genes involved in the indirect pathway for sucrose absorption. cwINV, cell-wall invertase; PLT, polyol transporter; STP, sugar transport protein. B: Genes involved in the direct sucrose absorption and breakdown pathway. nINV, neutral/alkaline invertase; SuSy, sucrose synthase; UGPP, UDP-glucose pyrophosphorylase. C: Genes encoding plastid envelope transporters. All expression data were transformed to log2 scale for plotting the profiles. GPT, Glc-6-P/phosphate translocator; NTT, ATP/ADP transporter (Adenine nucleotide translocase); PEP, phosphoenolpyruvate; PPT, phosphoenolpyruvate/phosphate translocator; TPT, triose phosphate/phosphate translocator; XPT, xylulose 5-phosphate/phosphate translocator. Values less than -2 indicate that no expression of the gene was detected at the indicated point. Genes shown in this figure are listed in Table S2.

Sucrose is either directly transported into sink cells by the sucrose transporter (SUT) or, following its cleavage by an extracellular invertase (cwINV), the monosaccharides glucose and fructose are taken up by sugar transport proteins (STPs) and polyol transporters (PLTs) [28]–[29]. In cells, sucrose cleavage can be catalyzed by sucrose synthase (SuSy) and intracellular invertases (vacuolar invertases, vINV, or neutral/alkaline invertases, nINV). Physic nut has five putative cell wall-type invertase (cwINV) genes, eighteen putative *STP* and seven putative *PLT* genes, according to alignments with Arabidopsis and poplar orthologs [29]–[30]. Expression levels of two *cwINV* genes (JC_C100020497 and JC_C100025743), three *STP* genes (JC_C100009119, JC_C100014694 and JC_C100026553) and two *PLT* genes (JC_C100027064 and JC_C100007576) are highest before the filling stage, while those of three *PLT* genes (JC_C100000725, JC_C100025470 and JC_C 100025471) are highest during the filling stage in the seeds (Figures 3A and S1). Physic nut has only three *SUT* genes, all expressed at relatively high levels in developing seeds, but the expression level of two decreased during the filling stage. Physic nut has six *SuSy* genes: one (JC_C100010353) maximally expressed in the filling stage, and two (JC_C100024259 and JC_C100025110) that are strongly expressed but their expression level declines in the filling stage. The expression level of *vINV* genes also declines during the filling stage, while expression levels of seven of the eight *nINV* genes remain high in the filling stage (Figures 3B and S1). These results suggest that different sugar importing pathways are active during different developmental stages in physic nut seeds.

Sugar-phosphates transported across the plastid envelope membrane by phosphate/glucose-6-phosphate transporter (GPT), phosphate/phosphoenolpyruvate translocator (PPT), phosphate/triose-phosphate transporter (TPT), and xylulose-5-phosphate/phosphate translocator (XPT) [31]–[33]. Another key enzyme in translocation and assimilation processes is the plastidic ATP/ADP transporter (NTT), which plays a crucial role in supplying storage plastids with ATP [34]–[35]. GPT, PPT, and TPT are each encoded by two genes, while XPT and NTT are both encoded by a single gene in the physic nut genome. Expression levels of *GPT* (JC_C100019452), *TPT* (JC_C100026660), *PPT* (JC_C100021622) and *NTT* (JC_C100013456) genes remained high during the entire seed development process and peaked at S3–S5, while the *XPT* gene (JC_C100014296) was expressed at a relatively low level in the seeds (Figures 3C and S1).

### Central Metabolism-related Genes

**Figure 4:**
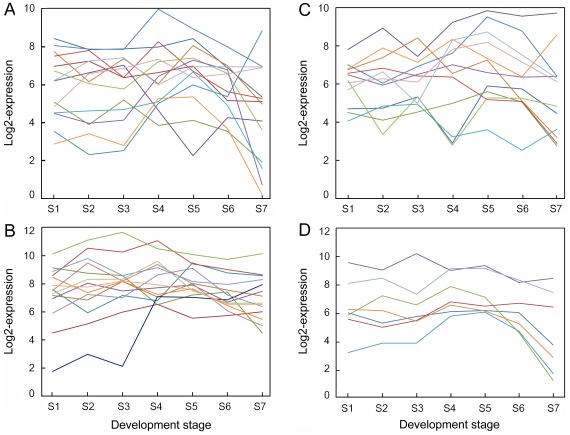
Overview of expression profiles of central metabolism pathway genes in developing physic nut seeds. A: Profiles of plastid glycolysis metabolic pathway genes. B: Profiles of cytosol glycolysis metabolic pathway genes that are highly expressed at the filling stage. C: Profiles of oxidative pentose phosphate pathway (OPPP) genes. D: Profiles of C3–C4 intermediary metabolism pathway genes. All expression data were transformed to the log2 scale for plotting the profiles. Lines in different colors indicate the expression patterns of different genes in the metabolic pathway. Genes shown in this figure are listed in Table S2.

The glycolysis pathway, oxidative pentose phosphate pathway (OPPP), and C3–C4 intermediary metabolism pathway have been suggested to generate glycerol-3-phosphate and NAD(P)H for FA and TAG synthesis in oilseeds [36]–[39]. Many enzymes involved in these metabolic pathways are encoded by multiple genes in the physic nut genome. As shown in Figure S2, at least one gene encoding an enzyme catalyzing each step involved in the glycolysis pathway and OPPP was highly expressed during the entire course of seed development. Our data confirm that genes encoding both plastid (Figure 4A) and cytosol (Figure 4B) enzymes of the pathway are expressed at high levels throughout seed development, although the expression of some genes, especially the plastid pathway genes, declined during the late maturing stage, S7. On the other hand, the expression levels of most genes encoding cytosolic glycolytic enzymes were higher than those encoding the plastidial isoforms in the developing seeds. This result is consistent with observations that ESTs for cytosolic glycolytic enzymes are more abundant than those of the plastidial isoforms in developing seeds of several oilseed plants [40]. The expression level of most OPPP enzyme genes (Figure 4C) and C3–C4 intermediary metabolism pathway genes (Figure 4D) peaked at the filling stages, S4 to S6, in the developing physic nut seeds.

### Starch Metabolism, Fatty Acid, TAG and Oil-body Biosynthesis Genes

**Figure 5:**
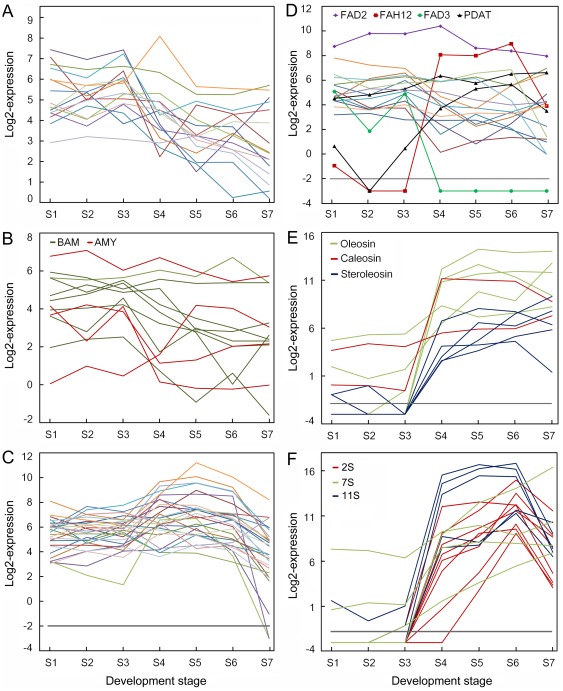
Overview of expression profiles of genes involved in starch metabolism and reserve synthesis in developing physic nut seeds. A: Profiles of starch synthesis pathway genes. B: Profiles of α-amylase (AMY) and β-amylase (BAM) starch breakdown genes. C: Profiles of fatty acid synthesis pathway genes. D: Profiles of triacylglycerol assembly pathway genes. FAD2, oleate desaturase; FAD3, linoleate desaturase; FAH12, oleic acid hydroxylase; PDAT, phospholipid:diacylglycerol acyltransferase. E: Profiles of oil-body protein genes. F: Profiles of seed storage protein genes. 11S, homologs of 11S globulins; 2S, homologs of 2S albumins; 7S, homologs of 7S and 7S-like globulins. All expression data were transformed to log2 scale for plotting the profiles, and lines in different colors in A, C, and D indicate the expression patterns of different genes in the respective metabolic pathway. Values less than −2 indicate that no expression of the gene was detected at the indicated point. Genes shown in this figure are listed in Table S2.

ADP-glucose pyrophosphorylase (AGPase) catalyzes the first step in starch biosynthesis, yielding the activated glucosyl donor ADP-glucose [41]. It comprises two large subunits (AGPL) and two small subunits (AGPS), each of which is encoded by distinct genes. The AGPS1 (JC_C100009747), AGPL1 (JC_C100011490) and AGPL2 (JC_C100015930) genes were all expressed in developing seeds, but at lower levels at S5 and S6. The expression level of most starch synthesis genes peaked at S3, then declined during the filling stages from S4 or S5 (Figures 5A and S3). The different starch degradation enzyme genes had different expression patterns in the developing seeds (Figures 5B and S3). Two α-amylase genes (JC_C10008238 and JC_C100011196) and two β-amylase genes (JC_C100014576 and JC_C 00002332) were strongly expressed during the filling stages, S4 to S6, while six β-amylase genes and two α-amylase genes were highly expressed in the early developing stage (Figures 5B and S3).

Most genes of the fatty acid synthesis pathway [10]–[11] were expressed at relatively high levels from S4 to S6, and their expression peaked at S5 in the developing seeds (Figures 5C and S4), except for acyl-ACP thioesterase B (*FATB*, JC_C100013836), expression of which decreased at the filling stage. The expression profiles of TAG assembly pathway genes are shown in Figures 5D and S5. At least one gene encoding an enzyme catalyzing each step of the pathway was highly expressed at the filling stage of the seeds (Figure S5). Of the phosphatidylcholine cycle genes, the oleate desaturase coding gene (*FAD2*, JC_C100004186) was highly expressed in the developing seeds, the oleic acid hydroxylase coding gene (*FAH12*, JC_C100003840) was expressed at the filling stage, but no linoleate desaturase coding gene (*FAD3*, JC_C100006612) transcripts were detected in filling stage seeds (Figures 5D and S5). Two phospholipid:diacylglycerol acyltransferase genes (JC_C100025621 and JC_C100030570) involved in TAG formation were highly expressed at the filling stage (Figure 5D). Expression of most oil-body protein genes began or increased from S4 and remained high during seed maturation (Figures 5E and S5). The expression of several fatty acid synthesis genes, from early development to the point where the dry weight of seeds peaks, has been analyzed by Xu et al. [26]. In accordance with their observations, we found that expression of the β-ketoacyl-ACP synthase I (KASI) gene increased before those encoding KASII and KASIII. In contrast, the expression of genes encoding glycerol-3-phosphate dehydrogenase, glycerol-3-phosphate acyltransferase, 1-acylglycerol-3-phosphate acyltransferase, acyl-CoA: diacylglycerol acyltransferase, phosphatidate phosphatase, which are involved in TAG assembly, did not match the profiles for genes involved in FA synthesis (Figures 5D and S5) as observed in several oilseed plants [40].This supports the suggestion that TAG accumulation may not require coordinated expression of acyltransferase transcripts and/or may involve post-transcriptional regulation [40].

### Seed Storage Protein and Late Embryogenesis-abundant Protein Genes

The storage proteins in seeds are important sources of amino acids in animal feed. We examined the gene models that showed similarity to known genes encoding seed storage proteins and their expression patterns in developing physic nut seeds. The draft of the physic nut genome reveals five 11S globulin (Legumin), three 7S globulin (Vicilin), two Vicilin-like and eight 2S albumin genes (Figure S6). The expression patterns of these storage protein genes were examined and compared to those of late embryo abundant (LEA) protein genes [42]. The expression level of 11S globulin genes and 2S albumin genes were highest at S6, while those of 7S globulin genes and most LEA protein genes were highest at S7 (Figures 5F and S6). These results indicate that expression levels of 11S globulin genes and 2S albumin genes peak before those of 7S globulin genes and LEA protein genes in developing physic nut seeds.

### Transcription Factors Function in Storage Compound Biosynthesis and Accumulation in the Seeds

**Figure 6:**
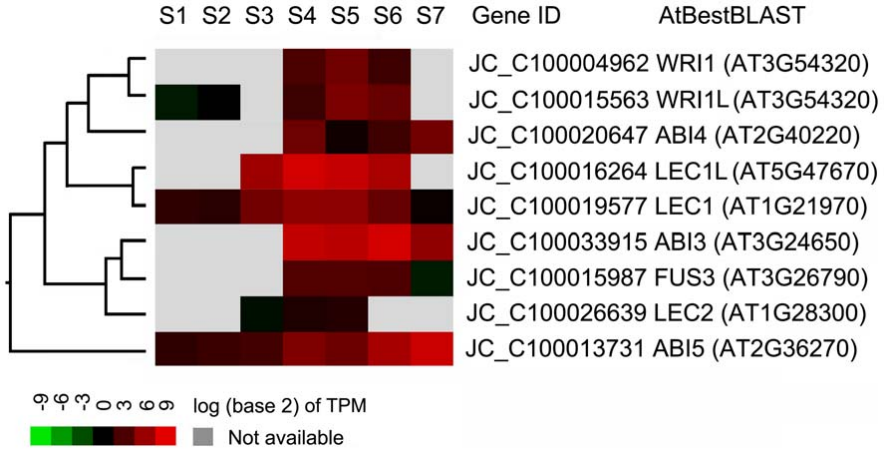
Expression profiles of transcription factor homologues corresponding to those known to regulate reserve synthesis in Arabidopsis.

At least 114 transcription factor (TF) genes were co-expressed with seed storage reserve synthesis genes during seed maturation, including orthologs of identified TFs that play important roles in seed reserve synthesis in Arabidopsis [11], [20], such as LEC1 (JC_C100019577), LEC1L (JC_C100016264), FUS3 (JC_C100015987), LEC2 (JC_C100026639), WRI1 (JC_C100004962 and JC_C100015563), ABI3 (JC_C100033915), ABI4 (JC_C100020647) and ABI5 (JC_C100013731). Several of these TFs are embryogenesis-related genes, such as PEI1 (JC_C100018497), AtMYB118 (JC_C100013351), bHLH95 (JC_C100022524) and AGL15 (JC_C100012112) (Table S3). In the physic nut developing seeds, the *LEC1*, *WRI1L*, and *ABI5* genes were expressed from the earliest stage, *LEC2* and *LEC1L* genes were expressed from S3, while *FUS3*, *ABI3*, *ABI4*, and *WRI1* genes were expressed from S4 (Figure 6).

## Discussion

Studies on the regulation of physic nut lipid metabolism have important fundamental and practical applications. Here, we provide a systematic analysis of gene expression profiles in developing physic nut seeds. The work has also revealed global insights into the regulatory network of FA metabolism of the physic nut seed, and will thus be helpful for oil-crop engineering.

### 29–41 DAP is the Key Period for Seed Storage Reserve Synthesis in Physic Nut Seeds

The histodifferentiation and accumulation of dry matter in the seed coat of the physic nut seeds occurred before 29 DAP, under our field conditions (Figure 1A). Rapid increase of the kernel dry mass started at 29 DAP and ended at 41 DAP (Figure 1A). During this period, the starch content declined from ca. 11% to ca. 2%, while the lipid content increased from ca. 3% to ca. 55% of kernel dry weight (Figure 1D), the storage protein level increased (Figure 1C) and the fatty acid composition significantly changed (Figure 2C). Expression analysis revealed that opposite shifts in expression levels of starch and fatty acid synthesis genes also occurred from 29 to 41 DAP (Figure 5A and 5C, respectively). Oil-body protein genes (Figure 5E) and seed storage protein genes (Figure 5F) were highly expressed at this stage. Thus, 29–41DAP is the critical seed storage reserve synthesis and accumulation period in developing physic nut seeds. In addition, the crucial period for the transition of seed-to-sink tissue was around 29 DAP. At this point, expression of several seed storage reserve synthesis regulation genes, such as *WRI1*, *FUS3*, and *ABI3* began (Figure 6), the expression of starch synthesis genes decreased (Figure 5A) and that of fatty acid synthesis genes increased (Figure 5C).

### Sucrose Import and Breakdown

Sucrose import and breakdown are important processes for seed production and development. In *B. napus* L. seeds, the expression levels of *SUT1* control some of the phenotypic variation for branch number and seed weight [43]. The rice *GIF1* (*GRAIN INCOMPLETE FILLING 1*) gene encodes a cell-wall invertase required for carbon partitioning during early grain-filling [44]. The acid invertases are targeted to the cell wall (cwINV) and vacuole (vINV), neutral/alkaline invertases (nINV) function in the cytosol [45], and the monosaccharides glucose and fructose are taken up by sugar transport proteins (STP) and polyol transporters (PLT) [29]–[30]. In our developing physic nut seeds, cwINV genes were highly expressed at the early filling stage (Figures 3A and S1). The STP and PLT genes were also expressed, at low levels, at the late filling stage (Figures 3A and S1). In contrast, the total expression level of sucrose transporter (SUT), sucrose synthase (SuSy) and nINV genes was high not only during the early developing stage but also during the filling stage (Figures 3B and S1). These results imply that the direct sucrose importing pathway plays the major role in sucrose import, while the monosaccharide importing pathway plays secondary role during the filling stage in developing physic nut seeds.

Phosphoenolpyruvate and triose-phosphate are the key carbon sources imported into the plastid, by the phosphoenolpyruvate translocator (PPT) and triose-phosphate translocator (TPT), respectively, for FA synthesis, while the glucose-6-phosphate (Glc-6-P) required for starch biosynthesis and the oxidative pentose phosphate pathway (OPPP) is imported by the Glc-6-P transporter (GPT). Mutation of GPT1 alters the expression of genes involved in starch turnover and the oxidative pentose phosphate pathway, and results in seed abortion at the end of the globular stage of embryo development, in Arabidopsis [46]. In the *PPT1 Arabidopsis* mutant *cue1*, the oil content of mature seeds is reportedly 10 to 20% lower than in wild type [47]. The xylulose 5-phosphate/phosphate translocator (XPT) accepts xylulose 5-phosphate, ribulose 5-phosphate and erythrose 4-phosphate as counter exchange substrates for Pi between the plastid and cytosol [33]. In addition, the nucleoside triphosphate transporter (NTT) provides the plastid stroma with ATP from the cytosol. Arabidopsis possesses two NTTs, NTT1 and NTT2, with similar transport characteristics. Loss of NTT2, but not NTT1, impairs the supply of cytosolic ATP to plastids, resulting in a reduced seed oil phenotype [34]–[35]. In both Arabidopsis and *B. napus*, PPT-encoding genes are reportedly expressed strongly at all stages of seed development, including later stages, while *GPT* genes are expressed at high levels during early stages [8], [48]. Genes encoding GPT (JC_C100019452), PPT (JC_C100021622) and TPT (JC_C100026660) were all strongly expressed during all stages of physic nut seed development until maturation (Figures 3C and S1). The NTT gene was also strongly expressed, while the XPT gene was weakly expressing during development of the seeds (Figure 3C). These results indicate that C3 and C6 sugar phosphates, but not C4 and C5 sugar phosphates, are the major sugar phosphates imported into plastids in developing physic nut seeds. The results also imply that the *GPT*, *PPT*, *TPT* and *NTT* genes play important roles in importing sugar and energy into plastids for fatty acid synthesis in physic nut seeds.

### Activities of Three Sugar Metabolic Pathways in Developing Physic Nut Seeds

The hexose phosphates generated by sucrose cleavage can be metabolized through glycolysis or the oxidative pentose phosphate pathway (OPPP). This central pathway is a key component of a metabolic network, providing inputs of precursors for fatty acid and TAG synthesis, namely glycerol-3-phosphate and NAD(P)H. Each of the glycolysis enzymes is present in both the cytosol and plastids, and both the cytosolic [49]–[50] and plastidial glycolytic [36]–[37], [39], [48], [51] pathways have been suggested to play central metabolic roles during oilseed development. The role of the OPPP in fatty acid production has been described in several reports [52]–[54]. OPPP enzymes have been detected in proteomic analyses of filling castor seeds [36], while metabolic feeding of purified organelles and in vivo metabolic flux analyses strongly implicate involvement of the C3–C4 intermediary metabolism pathway in castor bean and soybean filling, respectively [55]–[56]. In the latter pathway, phosphoenolpyruvate can be carboxylated to oxaloacetate by phosphoenolpyruvate carboxylase (PEPC). Expression levels of most genes of the cytosolic and plastidial glycolysis, OPPP and C3–C4 intermediary metabolism pathways remained high during the S4 to S6 stages, but those of all but the cytosolic glycolysis pathway declined at S7 (Figure 4), when oils rapidly accumulate in developing physic nut seeds (Figures 1C and 5C). The decline in expression of genes of the plastid glycolysis, OPPP and C3–C4 intermediary metabolism pathways is consistent with a low rate of oil accumulation at the S7 stage. Hence, our results strongly suggest that these three metabolic pathways play important roles in oil synthesis in physic nut seeds, while the continuing high expression of most genes of the cytosolic glycolysis pathway during S7 indicates that cytosolic glycolysis plays other roles during the maturation stage of the seeds.

### Starch Metabolism in Developing Physic Nut Seeds

In oilseed plants such as *B. napus* and Arabidopsis, starch deposition occurs transiently in the early stages of embryo development. Starch accounts for approximately 8% to 10% of the seed dry weight in these stages, and is absent in mature seeds [57]–[58]. Phosphoglucomutase, which catalyzes the readily reversible interconversion of Glc-1-P and Glc-6-P, is essential for starch synthesis and plays an essential role in the degradation of assimilatory starch. Seeds of plastidial phosphoglucomutase gene knockdown mutants reportedly accumulate 40% less oil than wild type Arabidopsis seeds, indicating that starch is positively linked to oil accumulation in seeds [59]. Further, ADP-Glc pyrophosphorylase (AGPase) catalyzes the first step in the starch biosynthesis pathway, and knockdown of the small subunit of AGPase reduced the starch content and rate of starch synthesis, with a consequent 50% reduction in lipid contents, in *B. napus* seeds examined by Vigeolas et al. [60]. The starch content is ca. 11% of the dry weight in early stage kernels and, following a net reduction in starch content of the seeds after S5 (Figure 1C, 1D), ca. 2% in kernels of mature physic nut seeds. Accordingly, starch synthesis genes and some starch breakdown genes were highly expressed before S5 in the seeds (Figures 5A and S3). In contrast, several starch breakdown genes were highly expressed at the filling stage (Figures 5B and S3). These results suggest that starch is actively turned over during early stages of physic nut seed development. During the filling stage, the starch synthesis rate declined, while the starch breakdown rate remained high, resulting in a net reduction of starch levels in the seeds.

### Changes in the Fatty Acid Composition of Physic Nut Oils are Consistent with Relative Expression Levels of Different Types of Acyl-ACP Thioesterase and Fatty Acid Desaturase Genes

The ability of a biological resource to meet specified criteria for use as a biodiesel fuel is largely determined by its FA composition [61]–[63]. Thus, identification of means to manipulate the FA composition of candidate resources would be highly valuable, and the coinciding changes in FA composition and expression patterns of genes involved in FA biosynthesis and modification in developing physic nut seeds provide a convenient model for exploring and potentially refining FA biosynthesis in plants. Physic nut oil (Figure 2B) contains more C18∶1, but less C18∶3 and C20, than Arabidopsis oil [64]. In Arabidopsis seeds the fatty acid elongase 1 (FAE1, At4G34520) of the TAG synthesis pathway is required for synthesis of very long chain fatty acids [65], the *FAD2* gene encodes an endoplasmic reticulum 18:l desaturase [66], while the *FAD3* gene encodes an endoplasmic reticulum 18∶2 desaturase [67]. We detected no FAE1 subfamily homologous gene in the physic nut genome, and no *FAD3* transcripts in physic nut seeds at the filling stage (Figures 5D and S5), although *FAE1* and *FAD3* genes are reportedly highly expressed during this stage in Arabidopsis seeds [8].

Acyl-ACP thioesterase A (FATA) has a high specificity for 18∶1-ACP and a lower activity toward 18∶0- and 16∶0-ACP, whereas, FATB more efficiently catalyzes the reaction with ACPs carrying saturated fatty acyl chains [68]–[69]. During the development of the physic nut seed, the content of C18∶1 increased while C16∶0 and C18∶0 contents decreased (Figure 2C). In accordance with the changes in levels of these fatty acids, the expression level of stearoyl-ACP desaturase (SAD) genes and *FATA* increased, whereas *FATB* expression decreased in filling stage seeds (Figure S4). Overall, the fatty acid composition of the oil in the developing physic nut seeds was consistent with the relative expression levels of different isoforms of both acyl-ACP thioesterase and fatty acid desaturase genes. Thus, differences in the expression profiles of FAE1 and fatty acid desaturase genes appear to make major contributions to the differences in fatty acid composition of TAGs between physic nut and Arabidopsis oils. This information provides valuable indications of possible ways to optimize the FA composition of oils in seeds of both physic nut and other oilseed plants in order to improve their fuel properties using genetic and transgenic methods.

About 90% of Castor bean oil consists of ricinoleate (12-hydroxy-oleate), produced from oleoyl-phosphatidycholine by the catalytic activity of a FAD2 family protein, oleic acid hydroxylase (FAH12) [70]. The expression patterns of fatty acid synthesis genes and *FAH12* in developing physic nut seeds (Figures 5C–D, S4 and S5) were similar to those in developing castor bean seeds [70], except that the FAD2 gene was highly expressed in the physic nut seeds, but is very weakly expressed in castor bean endosperm [71]. A castor bean phospholipid:diacylglycerol acyltransferase (PDAT) gene, RcPDAT1A, also specifically enhances the reaction that generates ricinoleate in vivo and generates castor-like TAGs in *RcFAH*-expression Arabidopsis (CL37 parental line) seeds [72]–[73]. The physic nut genome has both a *FAH12* gene (JC_C100003840) and a *PDAT1A* gene (JC_C100025621). Further, the presence of ricinoleate in its seed oil suggests that the physic nut FAH12 family gene probably encodes an active oleic acid hydroxylase (Figure 2A). However, physic nut seed oil contains very little ricinoleate (no more than 2% of the total FAs) (Figure 2B), although both the *FAH12* and *FAD2* genes were highly expressed at the filling stage of physic nut seeds (from S4 to S6) (Figures 5D and S5). The hydroxy fatty acid content (total HFA) is reduced from ca. 17% in the Arabidopsis CL37 parental line to less than 5% in the most-extreme *FAD2* transgenics [73]. Thus, transcriptional regulation of *FAD2* and *FAH12* genes may be one of the mechanisms that contribute to a high level of ricinoleate accumulation in castor endosperm [73], and the low content of ricinoleate in physic nut oils may be due to the high expression of the *FAD2* gene in the seeds. Alternatively, the physic nut FAH12 enzyme may have lower oleic acid hydroxylation activity and the PDAT1A enzyme lower specificity for ricinoleate than the corresponding castor bean enzymes. Hence, further study of the enzymatic activities and substrate specificities of physic nut FAH12 and PDAT1A enzymes should provide interesting information on the differences in evolution and roles of lipid synthesis enzymes between physic nut, castor bean and other oilseed plants.

### Storage Protein Genes Shown Different Expression Patterns in Developing Physic Nut Seeds

Seed storage proteins in seeds are important source of amino acids for animal feed. The protein composition of physic nut seed meal has been analyzed, and shown to compare favorably with soybean meal, containing a good balance of essential amino acids, with the exception of lysine [74]–[75]. Physic nut seed storage proteins, 11S globulins and 2S albumins, but not 7S globulins, contain less Lys than soybean proteins according to the deduced amino acids of their coding genes (Table S4). The expression of the 11S globulin and 2S albumin genes began before the 7S globulin genes during filling of the physic nut seeds (Figures 5F and S6), and their longer duration of high level expression in the developing seeds is likely to be an important contributor to the relatively low Lys content of the mature seeds’ proteins.

### Conservative Transcriptional Regulation between Physic Nut and Arabidopsis Seed Reserve Accumulation

In previous Arabidopsis studies a model for coordinated transcriptional regulation of the synthesis of seed storage reserves has been proposed [11], [20]. The present study provides the first profiling of transcription factors throughout the development of physic nut seeds. The detected genes include many putative orthologs of genes involved in embryogenesis and seed reserve synthesis in Arabidopsis according to amino acid sequence alignments. In Arabidopsis, the transcription factor WRINKLED1 (WRI1) is involved in the regulation of seed storage metabolism, and expression of genes involved in glycolysis and lipid synthesis in developing seeds is compromised in *wri1* mutants [11]–[12]. WRI1 functions downstream of LEC1 and LEC2, and ectopic expression of LEC1, LEC2 or WRI1 in vegetative tissues up-regulates the expression of genes involved in FA synthesis [13], [76]. Storage protein gene transcription is promoted by the combined action of LEC2, FUSCA3 (FUS3) and ABSCISIC ACID INSENSITIVE-3 (ABI3). The synthesis of these factors is in turn activated by LEC1 and LEC1L [19]–[20], [76]–[77], while ABI4 acts as a repressor of lipid breakdown in Arabidopsis seed germination [21]. At the end of seed filling, ABI5/EEL has been implicated in shutting down seed storage protein synthesis during seed desiccation, and activating several late embryogenesis-abundant genes, including *AtEm1* and *AtEm6*, during seed maturation [23]–[24], [78]. In developing physic nut seeds, the expression sequence of these genes is as follows: LEC1 gene at S1; LEC2 and LEC1L genes at S3; FUS2, ABI3, ABI4, and WRI1 genes at S4 (Figure 6; Table S3). As in Arabidopsis, this expression pattern of TF genes is consistent with the decreased expression of starch synthesis genes and increased expression of fatty acid and TAG synthesis and storage protein genes during the filling stages in the developing seeds (Figure 5C–F). The expression level of the ABI5 gene was highest at the last stage, S7, in accordance with the decline in expression of genes encoding 11S globulin and 2S albumin storage proteins, and up-expression of late embryo abundant protein genes (Figures 5F, 6 and S6). In contrast to Arabidopsis seeds, in which the storage compounds are deposited in cotyledons, physic nut seeds deposit storage compounds in the endosperm. These findings suggest that a similar regulatory network of TFs controls oil and seed storage protein accumulation in the endosperm of physic nut seed as in the embryos of Arabidopsis seeds. Thus, our data provide an important step toward identifying the regulatory gene networks responsible for programming the development of physic nut seeds. However, the reasons and roles of differences in the compartmentation of storage reserves (in endosperms of physic nut and cotyledons of Arabidopsis seeds) and seed-specific TF genes in this process remain to be determined.

In conclusion, the presented study of seed development, storage reserve accumulation and gene expression, provide integrative information for understanding the relationships of these processes during physic nut seed development. Our results show that the phase between 17 and 29 DAP is associated with rapid increases in seed volume and seed coat dry matter, while the seeds fill from 29 to 41 DAP during physic nut seed development (under our growth conditions). As proposed in Arabidopsis, the *WRI1*, *FUS3* and *ABI3*transcription factor genes were expressed from the onset of seed-filling (S4), which shifted the metabolic network towards the production of storage fatty acids and oils and seed storage proteins in the developing seeds. Expression levels of FATA, SAD and FAD2 genes were high, and the expression of FATB and FAD3 genes low in filling stage seeds, in accordance with the high contents of C18∶1 and C18∶2 lipids detected in physic nut seed oils. These data should facilitate understanding of the molecular basis of fatty acid and lipid biosynthesis, and identification of the key genes involved in regulation of the fatty acid composition, oil content and storage reserve biosynthesis during physic nut seed development. Further investigations of the functions of these genes and the scope for using them to manipulate the fatty acid composition and oil contents of seeds by genetic engineering means could substantially contribute to the refinement of biofuels.

## Materials and Methods

### Ethics Statement

No specific permits were required for the described field studies, because the experimental field is owned by South China Botanical Garden, Chinese Academy of Sciences, and the Key Laboratory of Plant Resources Conservation and Sustainable Utilization performs the management. No specific permits were required for these locations/activities, because the location is not privately-owned or protected in any way and the field studies did not involve endangered or protected species.

### Plant Materials and Chemicals

Physic nut (*J. curcas* L.) trees of the inbred cultivar GZQX0401 were grown in a natural environment on farmland in the South China Botanical Garden (Guangzhou, Guangdong Province, China). Seeds were sampled from artificially pollinated 4-year-old plants from 14 days after pollination (DAP) during the period of July-September, defining the pollination day as day 0. Thirty to one hundred seeds (after and before 24 DAP, respectively) were collected at each time point per biological replicate. For chemical analysis, about one hundred milligrams of dry seeds per biological replicate were ground to powder using a mortar and pestle. The seed oil was extracted and analyzed as described by Hara and Radin [79]. After removing the oil using acetone, protein was extracted by a previously described method [80] and quantified by the BCA method [81]. To extract starch, soluble sugars were first eluted from seed flour by 80% ethanol at 70°C. After centrifugation, the residual pellet was homogenized in 0.2 mol/L KOH and the suspension was incubated at 95°C for 1 h to dissolve the starch. Following the addition of 1 mol/L acetic acid and centrifugation for 5 min at 16 000 g, the starch content of the supernatant was assayed using the anthrone sulfuric acid method [82]. Assays were performed with three biological replicates.

The lipid composition of the samples was analyzed, following FAME derivatization, according to the method described by Li et al. [64], adding C17∶0 as an internal standard. For this analysis we used an Agilent 7890A gas chromatograph system equipped with a flame ionization detector (FID) and an HP-88 column (30 m×0.25 mm I.D., 0.20 µm film thickness), with chromatography conditions described by Wu et al. [83], coupled to a GCMS-QP2010 plus mass spectrometer (Shimadzu). The assay was performed with three biological replicates. The significance of between-sample differences in mean contents of the detected FAs was assessed using Student’s t test.

### Preparation of RNA

The seven developmental points were during: the early stage of seed development (histodifferentiation) (S1, 14 DAP; whole seeds), early increase of seed dry-weight stage (S2, 19 DAP; whole seeds), rapid increase of seed coat dry-weight stage (S3, 25 DAP; kernels), early increase of kernel dry-weight stage (S4, 29 DAP; kernels), rapid increase of kernel dry-weight stage (S5, 35 DAP; kernels), late kernel dry-weight increase stage (S6, 41 DAP; kernels), and maturation or desiccation stage (S7, 45 DAP; kernels) (Figure 1A, 1B). Total RNA was extracted from the samples using an RNeasy Plant Mini Kit (QIAGEN; Cat. No. 74904), and the isolated RNA was subsequently treated with RNase-Free DNase I (Roche, http://www.roche.com).

### Digital Gene Expression Library Preparation and Sequencing

Tag libraries of the samples of seeds in the seven developmental stages were prepared in parallel using an Illumina gene expression sample preparation kit and sequenced using the Illumina GAII platform at BGI-Shenzhen without replications (http://en.genomics.cn/navigation/index.action) (Methods S1). A preprocessed database of all possible CATG+17 nucleotide tag sequences was created using our genome reference database. Further information on the genomic sequences and the predicted protein-encoding genes is available at ftp://Jatropha:9uebluesrjd7@ftp.genomics.org.cn and at the NCBI nucleotide database (Project ID: 63485). For annotation, all tags were mapped to the reference sequences, allowing no more than one nucleotide mismatch per tag. All the tags that mapped to reference sequences from multiple genes were filtered and the remaining tags were defined as unambiguous tags. As a result, we generated between 3.26 and 6.18 million raw tags for each of the seven samples (Table S5). After removing the low quality reads, the total number of tags per library ranged from 3.03 to 6.07 million and the number of tag entities with unique nucleotide sequences ranged from 83,820 to 167,765 (Table S5). For gene expression analysis, the number of expressed tags was calculated and then normalized to TPM (number of transcripts per million tags) [27].

A 3×3 self-organizing map (SOM) of the gene expression data was constructed using GeneCluster 2.0 [84] (http://www.broadinstitute.org/cancer/software/genecluster2/gc2.html) with a variation filter (Max/Min≥5) to eliminate genes whose expression did not change significantly across samples, and normalization of the means and variance (mean  = 0 and variance = 1). The SOM algorithm was executed with the desired cluster range of 3–9 and the rest of the parameters left unchanged. They are 50000 iterations, seed range of 42, initialization of centroids to random vectors, bubble neighborhood, initial and final learning weights of.1 and .005, and initial and final sigmas determining the size of the update neighborhood of a centroid set to 5 and .5, respectively.

## Supporting Information

Figure S1 **Expression profiles of sucrose absorption and transporter genes.** Frc, fructose; Glc, glucose; GPT, Glc-6-P/phosphate translocator; INV, invertase; PEP, phosphoenolpyruvate; 3-PGA, -phosphoglycerate; PLT, polyol transporter; PPT, phosphoenolpyruvate/phosphate translocator; STP, sugar transport protein; Suc, sucrose; SuSy, sucrose synthase; SUT, sucrose transporter; TPT, triose phosphate/phosphate translocator; XPT, xylulose 5-phosphate/phosphate translocator; Xu-5-P, xylulose 5-phosphate.
(TIF)

Figure S2 **Expression profiles of central metabolism-related genes.** 1,3-BPG, 1,3-bisphosphoglycerate; DHAP, dihydroxyacetone-3-phosphate; E-4-P, erythrose-4-phosphate; Frc, fructose; GAP, glyceraldehyde-3-phosphate; Glc, glucose; 6-PG, 6-phosphogluconate; OAA, oxaloacetic acid; 6-PGL, 6-phosphogluconolactone; PEP, phosphoenolpyruvate; 2-PGA, 2-phosphoglycerate; 3-PGA, 3-phosphoglycerate; R-5-P, ribose-5-phosphate; PRPP, ribulose-1,5-bisphosphate; Ru-5-P, ribulose-5-phosphate; S-7-P, sedoheptulose-7-phosphate; UDP-Glc, uridine diphosphoglucose; Xu-5-P, xylulose-5-phosphate.
(TIF)

Figure S3 **Expression profiles of starch metabolism-related genes.** AGPL/S, ADP–glucose pyrophosphorylase large subunit/small subunit; AMY, alpha-amylase; BAM, beta-amylase; BE, starch branching enzyme; DPE, disproportionating enzyme; GBSS, granule-bound starch synthase; GWD, glucan water dikinase; ISA, isoamylase; MEX, maltose transporter; PHO, starch phosphorylase; PUL, pullulanase.
(TIF)

Figure S4 **Expression profiles of fatty acid synthesis-related genes.** Manual annotation according to acyl lipid metabolism genes in Arabidopsis (http://aralip.plantbiology.msu.edu/pathways and http://lipids.plantbiology.msu.edu/maps_list.htm?q=lipids/genesurvey/maps_list.htm). ACP, acyl carrier protein; BC, biotin carboxylase; BCCP, biotin carboxyl carrier protein; CT, carboxyltransferase; ER, enoyl-ACP reductase; FATA, acyl-ACP thioesterase A; FATB, acyl-ACP thioesterase B; HAD, hydroxyacyl-ACP dehydrase; HDLAP, dihydrolipoamide acetyltransferase of PDHC; HOM-ACCase, homomeric acetyl-CoA carboxylase (ACCase); KAR, ketoacyl-ACP reductase; KAS, ketoacyl-ACP synthase; LPD, dihydrolipoamide dehydrogenase; MCMT, malonyl-CoA: ACP malonyltransferase; PDHC, pyruvate dehydrogenase complex; SAD, stearoyl-ACP desaturase.
(TIF)

Figure S5 **Expression profiles of TAG synthesis and oil-body formation- related genes.** Manual annotation according to acyl lipid metabolism genes in Arabidopsis (http://aralip.plantbiology.msu.edu/pathways and http://lipids.plantbiology.msu.edu/maps_list.htm?q=lipids/genesurvey/maps_list.htm). ABCAT, ABC acyl transporter; ACBP, acyl CoA binding protein; CCT, choline-phosphate cytidylyltransferase; CK, choline kinase; DAG-CPT, diacylglycerol cholinephosphotransferase; DGAT, acyl-CoA:diacylglycerol acyltransferase; GPAT, glycerol-3-phosphate acyltransferase; GPDH, glycerol-3-phosphate dehydrogenase; FAD2, oleate desaturase; FAD3, linoleate desaturase; LACS, long-chain acyl-CoA synthetase; LPC, 1-acylglycerol-3-phosphocholine; LPAAT, 1-acylglycerol-3-phosphate acyltransferase; LPLAT, 1-acylglycerol-3-phosphocholine acyltransferase; PC, phosphatidylcholine; PDAT, phospholipid:diacylglycerol acyltransferase; PDCT, phosphatidylcholine:diacylglycerol cholinephosphotransferase; PLA2, phospholipase A2; PLA2A, phospholipase A2 activator; PP, phosphatidate phosphatase.
(TIF)

Figure S6 **Expression profiles of seed storage protein genes and late embryo abundance protein (LEA) genes.** SMP, seed maturation family protein.
(TIF)

Methods S1 **Supplemental Methods.**
(DOC)

Table S1 **Summary of statistical analysis of expression patterns of genes that were differentially expressed during physic nut seed development.**
(XLS)

Table S2 **Genes and their expression levels at the seven stages indicated in** **Figures 3** **,** **4** **, and** **5** **.**
(XLS)

Table S3 **Transcription factor genes expressed during seed storage reserve synthesis.**
(XLS)

Table S4 **Comparision of average essential amino acid contents of putative seed storage proteins in physic nut (Jc) and soybean (Gm).**
(XLS)

Table S5 **Distribution of clean tag copy numbers and sequencing saturation analysis.**
(XLS)
