# Supplementary figures and images for: Global Analysis of Gene Expression Profiles in Developing Physic Nut (Jatropha curcas L.) Seeds
Source: PLoS One. 2012 May 4;7(5):e36522. doi: 10.1371/journal.pone.0036522 (PMC3344900; doi:10.1371/journal.pone.0036522)

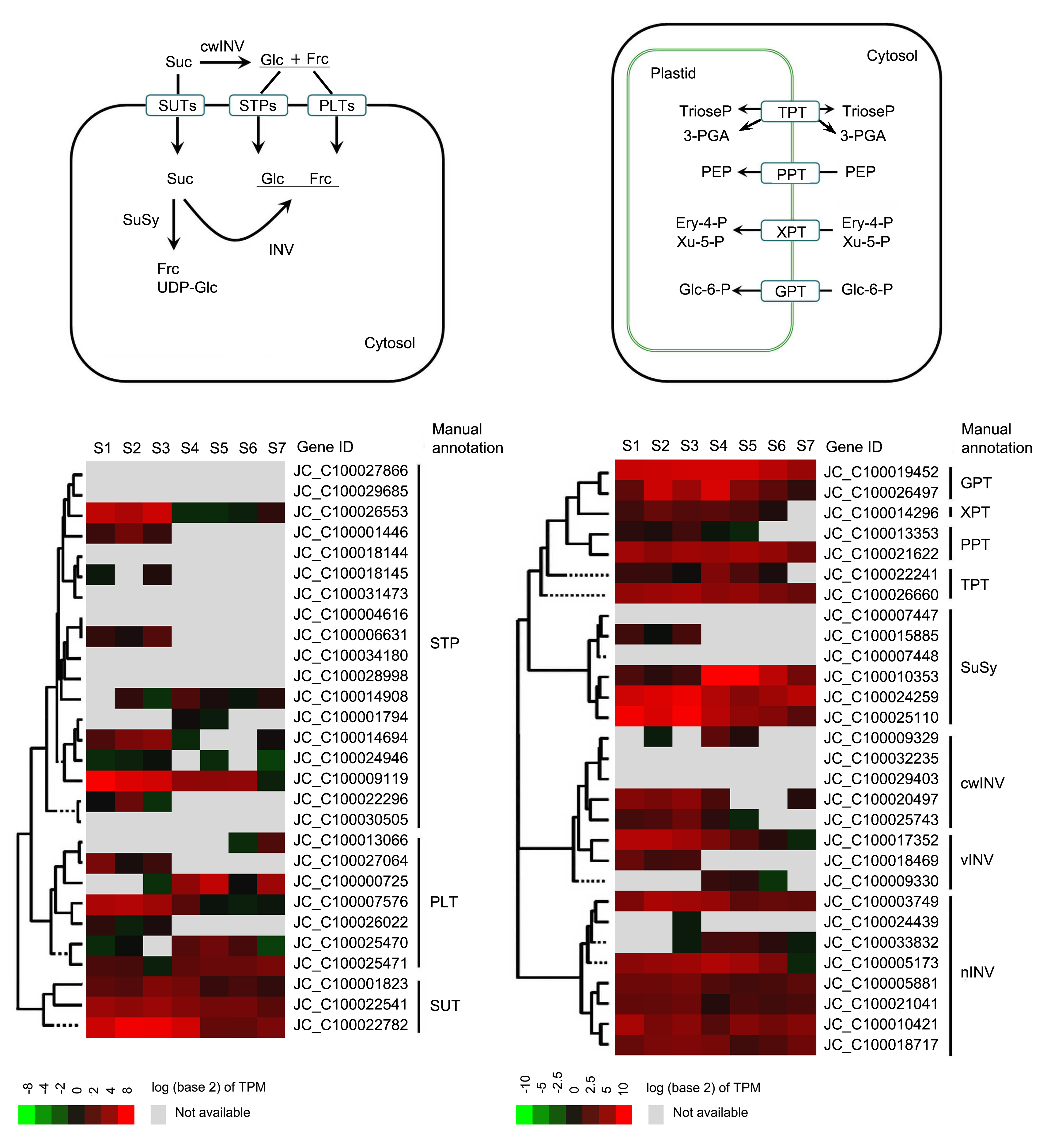

Supplement: Figure S1 — Expression profiles of sucrose absorption and transporter genes. Frc, fructose; Glc, glucose; GPT, Glc-6-P/phosphate translocator; INV, invertase; PEP, phosphoenolpyruvate; 3-PGA, -phosphoglycerate; PLT, polyol transporter; PPT, phosphoenolpyruvate/phosphate translocator; STP, sugar transport protein; Suc, sucrose; SuSy, sucrose synthase; SUT, sucrose transporter; TPT, triose phosphate/phosphate translocator; XPT, xylulose 5-phosphate/phosphate translocator; Xu-5-P, xylulose 5-phosphate. (TIF) [file pone.0036522.s001.tif]

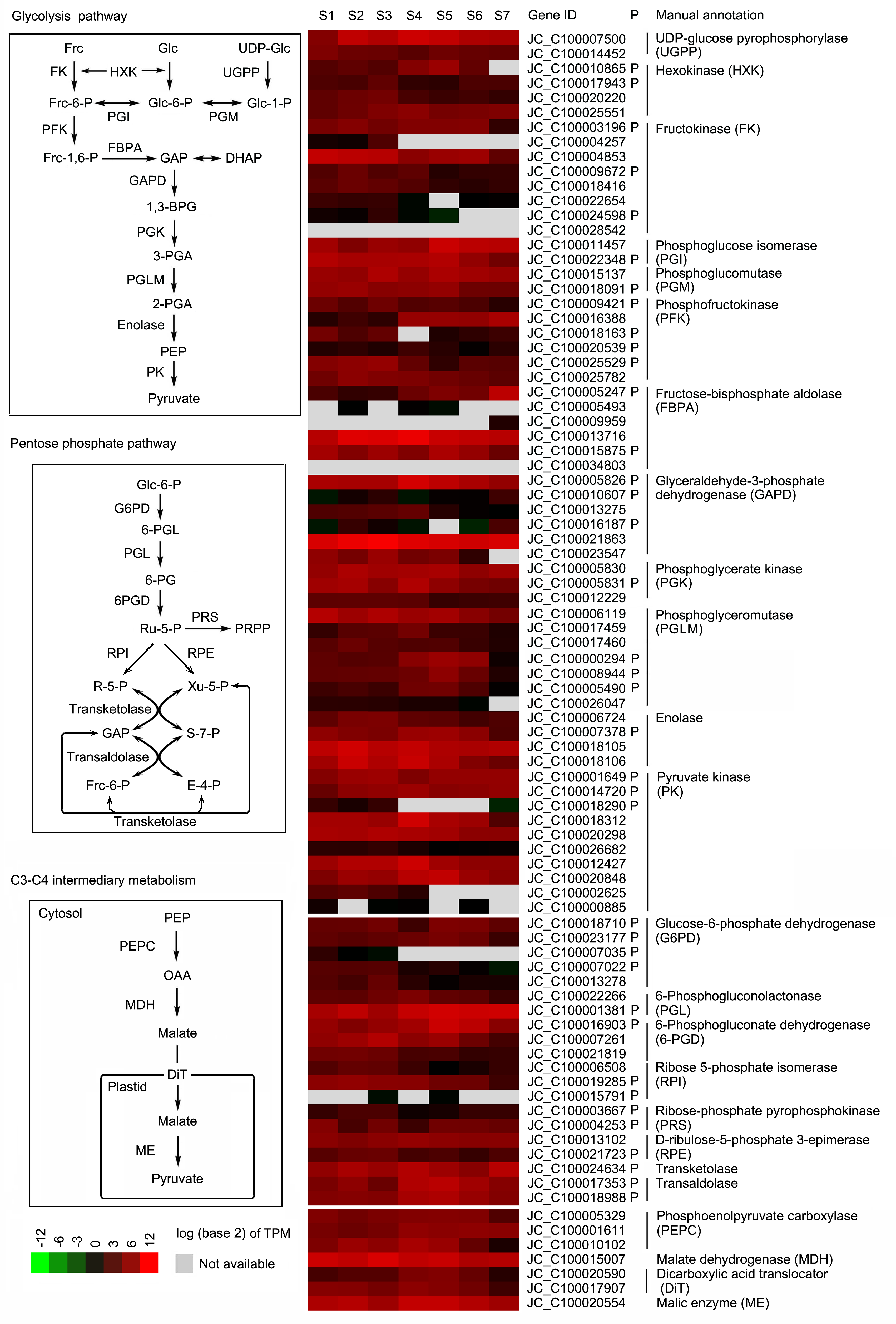

Supplement: Figure S2 — Expression profiles of central metabolism-related genes. 1,3-BPG, 1,3-bisphosphoglycerate; DHAP, dihydroxyacetone-3-phosphate; E-4-P, erythrose-4-phosphate; Frc, fructose; GAP, glyceraldehyde-3-phosphate; Glc, glucose; 6-PG, 6-phosphogluconate; OAA, oxaloacetic acid; 6-PGL, 6-phosphogluconolactone; PEP, phosphoenolpyruvate; 2-PGA, 2-phosphoglycerate; 3-PGA, 3-phosphoglycerate; R-5-P, ribose-5-phosphate; PRPP, ribulose-1,5-bisphosphate; Ru-5-P, ribulose-5-phosphate; S-7-P, sedoheptulose-7-phosphate; UDP-Glc, uridine diphosphoglucose; Xu-5-P, xylulose-5-phosphate. (TIF) [file pone.0036522.s002.tif]

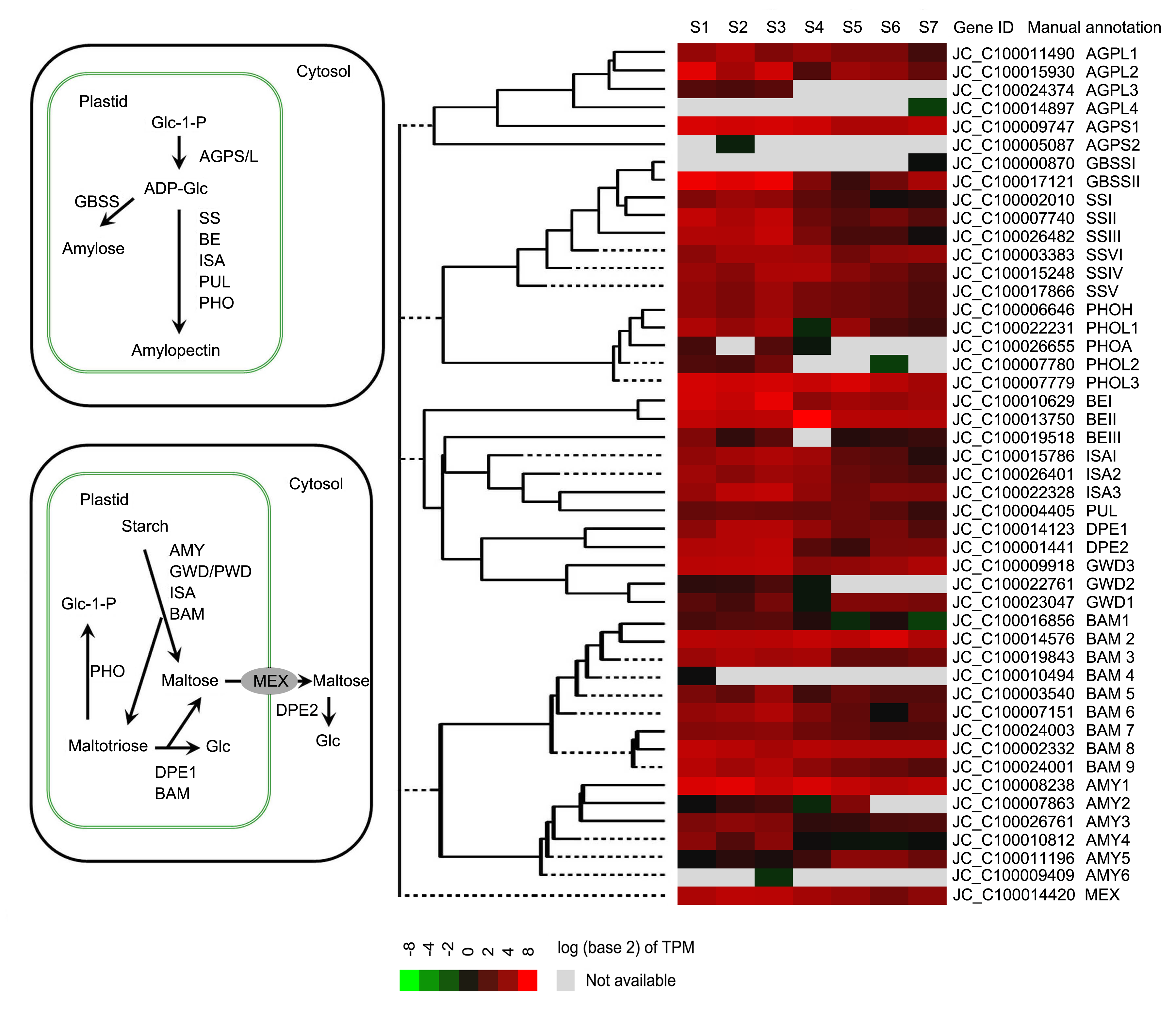

Supplement: Figure S3 — Expression profiles of starch metabolism-related genes. AGPL/S, ADP–glucose pyrophosphorylase large subunit/small subunit; AMY, alpha-amylase; BAM, beta-amylase; BE, starch branching enzyme; DPE, disproportionating enzyme; GBSS, granule-bound starch synthase; GWD, glucan water dikinase; ISA, isoamylase; MEX, maltose transporter; PHO, starch phosphorylase; PUL, pullulanase. (TIF) [file pone.0036522.s003.tif]

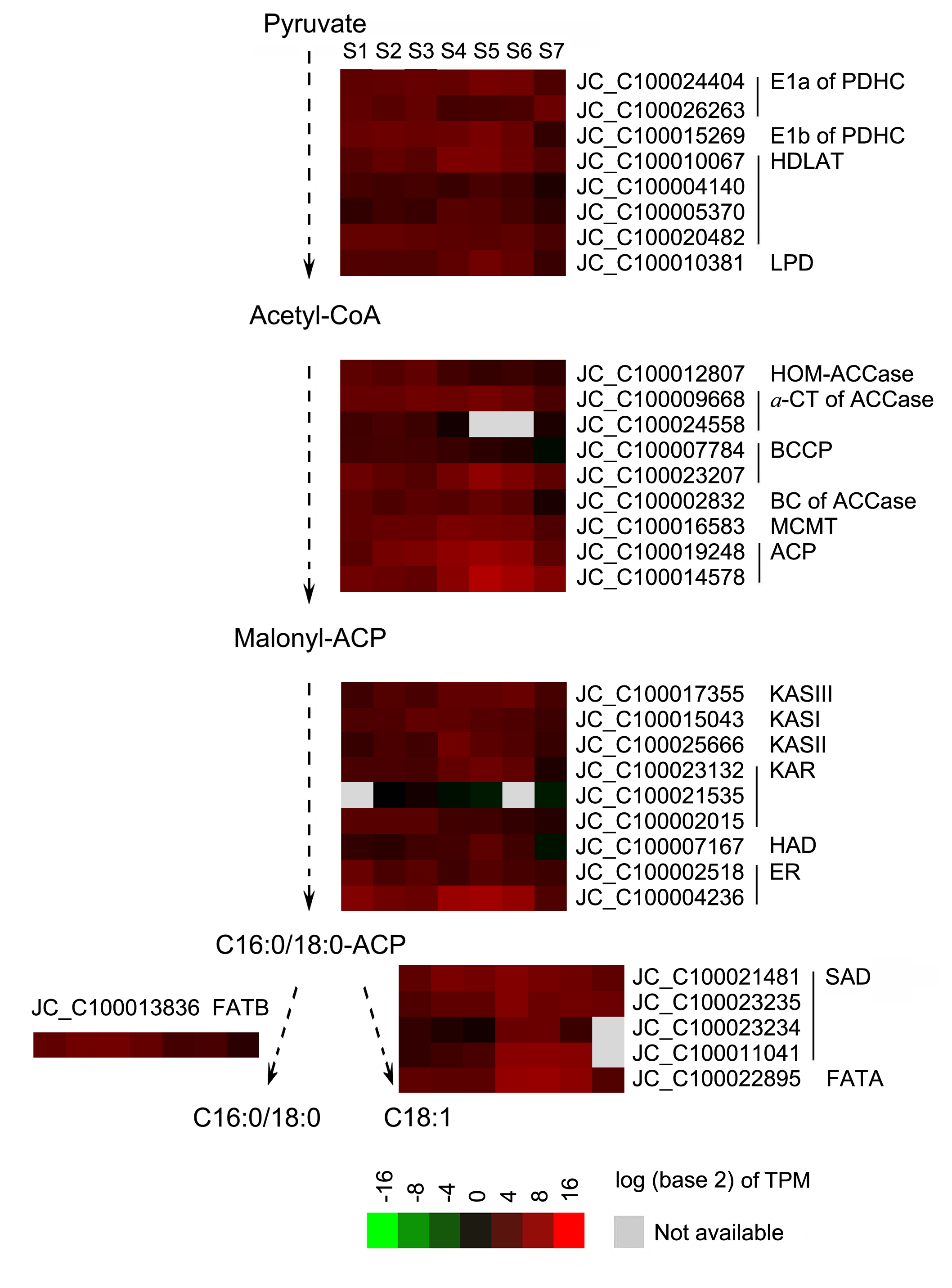

Supplement: Figure S4 — Expression profiles of fatty acid synthesis-related genes. Manual annotation according to acyl lipid metabolism genes in Arabidopsis (http://aralip.plantbiology.msu.edu/pathways and http://lipids.plantbiology.msu.edu/maps_list.htm?q=lipids/genesurvey/maps_list.htm). ACP, acyl carrier protein; BC, biotin carboxylase; BCCP, biotin carboxyl carrier protein; CT, carboxyltransferase; ER, enoyl-ACP reductase; FATA, acyl-ACP thioesterase A; FATB, acyl-ACP thioesterase B; HAD, hydroxyacyl-ACP dehydrase; HDLAP, dihydrolipoamide acetyltransferase of PDHC; HOM-ACCase, homomeric acetyl-CoA carboxylase (ACCase); KAR, ketoacyl-ACP reductase; KAS, ketoacyl-ACP synthase; LPD, dihydrolipoamide dehydrogenase; MCMT, malonyl-CoA: ACP malonyltransferase; PDHC, pyruvate dehydrogenase complex; SAD, stearoyl-ACP desaturase. (TIF) [file pone.0036522.s004.tif]

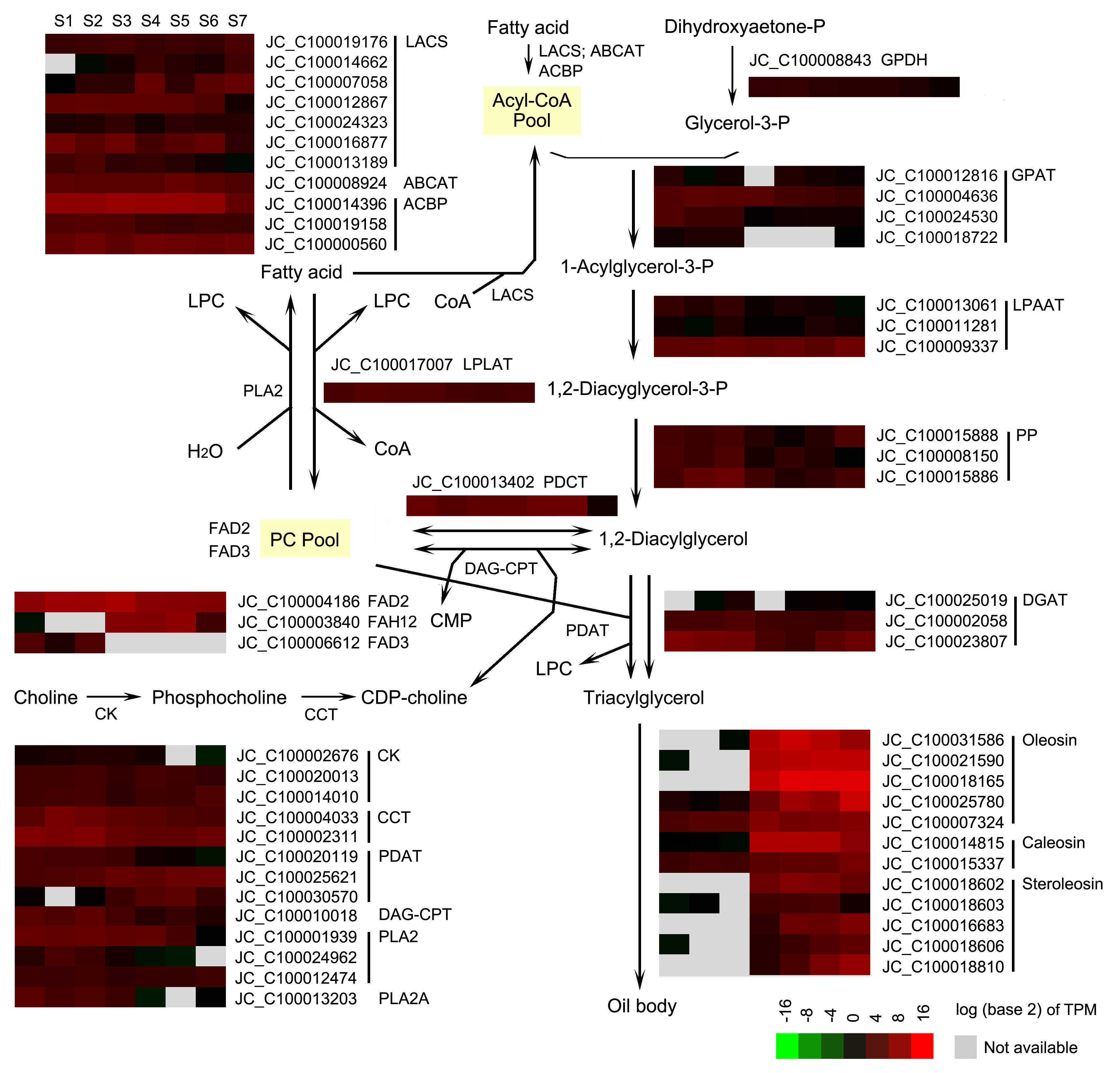

Supplement: Figure S5 — Expression profiles of TAG synthesis and oil-body formation- related genes. Manual annotation according to acyl lipid metabolism genes in Arabidopsis (http://aralip.plantbiology.msu.edu/pathways and http://lipids.plantbiology.msu.edu/maps_list.htm?q=lipids/genesurvey/maps_list.htm). ABCAT, ABC acyl transporter; ACBP, acyl CoA binding protein; CCT, choline-phosphate cytidylyltransferase; CK, choline kinase; DAG-CPT, diacylglycerol cholinephosphotransferase; DGAT, acyl-CoA:diacylglycerol acyltransferase; GPAT, glycerol-3-phosphate acyltransferase; GPDH, glycerol-3-phosphate dehydrogenase; FAD2, oleate desaturase; FAD3, linoleate desaturase; LACS, long-chain acyl-CoA synthetase; LPC, 1-acylglycerol-3-phosphocholine; LPAAT, 1-acylglycerol-3-phosphate acyltransferase; LPLAT, 1-acylglycerol-3-phosphocholine acyltransferase; PC, phosphatidylcholine; PDAT, phospholipid:diacylglycerol acyltransferase; PDCT, phosphatidylcholine:diacylglycerol cholinephosphotransferase; PLA2, phospholipase A2; PLA2A, phospholipase A2 activator; PP, phosphatidate phosphatase. (TIF) [file pone.0036522.s005.tif]

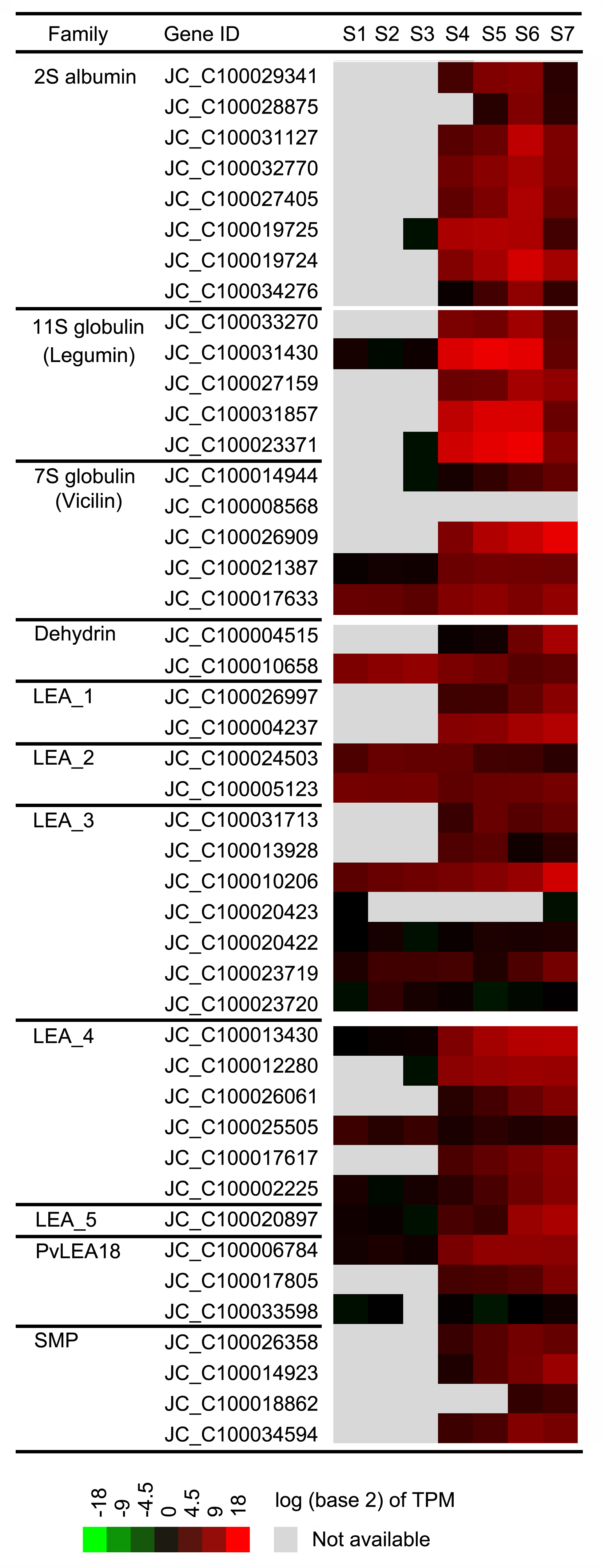

Supplement: Figure S6 — Expression profiles of seed storage protein genes and late embryo abundance protein (LEA) genes. SMP, seed maturation family protein. (TIF) [file pone.0036522.s006.tif]
