# Supplementary material for: Global Analysis of Gene Expression Profiles in Developing Physic Nut (Jatropha curcas L.) Seeds
Source: PLoS One. 2012 May 4;7(5):e36522. doi: 10.1371/journal.pone.0036522 (PMC3344900; doi:10.1371/journal.pone.0036522)
Supplement: Methods S1 — Supplemental Methods. (DOC) [file pone.0036522.s007.doc]

**Methods S1**

**Digital gene expression library preparation and sequencing**

The experimental process included sample preparation and sequencing. The main reagents and supplies were an Illumina Gene Expression Sample Prep Kit and Solexa Sequencing Chip (flow cell), and the main instruments were an Illumina Cluster Station and Illumina HiSeq™ 2000 System.

Specified Experimental Process: mRNA was purified from 6 μg of total RNA using oligo (dT) magnetic beads, then Oligo(dT) was used as primer to synthesize first and second-strand cDNA. The bead-bound cDNA was subsequently digested with the restriction enzyme *Nla*III, which recognizes and cuts off the CATG sites. The fragments, apart from the 3' cDNA fragments connected to Oligo(dT) beads, were washed away and the Illumina adaptor 1 was ligated to the sticky 5' end of the digested bead-bound cDNA fragments. The junction of Illumina adaptor 1 and CATG site is the recognition site of *Mme*I, a type of endonuclease with separated recognition and digestion sites. It cuts 17bp downstream of the CATG site, producing tags with adaptor 1. After removing 3' fragments by precipitation with magnetic beads, Illumina adaptor 2 was ligated to the 3' ends of tags, yielding tags with different adaptors at both ends to form a tag library. After 15 cycles of linear PCR amplification, 105 bp fragments were purified by 6% TBE PAGE. After denaturation, the single-chain molecules were fixed onto the Illumina Sequencing Chip.

In this system, each fixed molecule grows into a single-molecule cluster sequencing template through Situ amplification, four types of nucleotides, labeled by different colors, are then added and sequencing by synthesis (SBS) is performed. Each tunnel will generate millions of raw reads with a sequencing length of 49 bp.

**Filtering dirty tags**

Raw sequences have 3' adaptor fragments as well as a few low-quality sequences and several types of impurities. Raw sequences are transformed into Clean Tags after certain steps of data-processing. Data-processing steps:

1. 3' adaptor sequence removal: since Tags are only 21 nt long while the sequencing reads are 49 nt long, raw sequences have 3' adaptor sequences.

2. Empty reads removal (reads with only 3' adaptor sequences but no Tags).

3. Low quality Tags removal (Tags with unknown sequences 'N').

4. Removal of Tags that are too long or too short, leaving Tags 21 nt long.

5. Removal of Tags with a copy number of 1 (probably arising from sequencing error).

6. Generate Clean Tags.

**Distribution of clean tag copy number**

Heterogeneity and redundancy are two significant characteristics of mRNA expression. A small number of mRNA categories are highly abundant, while the majority is expressed at very low levels. The distribution of clean tag expression can be used to evaluate the normality of the whole data set. Total Clean Tags represent the sum of all clean tag number; Distinct Clean Tags represent all types of Clean Tags (Table S5).

**Gene expression annotation**

All clean tags were mapped to the reference sequences and only 1bp mismatch is considered. Clean tags mapped to reference sequences from multiple genes were filtered. The remaining clean tags were designed as unambiguous clean tags. The number of unambiguous clean tags for each gene was calculated and then normalized to TPM (number of transcripts per million clean tags). For manual annotation, protein sequences of physic nut involved in each metabolic pathway were firstly searched by BLASTP against our physic nut protein database using Arabidopsis proteins. Secondly, the retrieved physic nut proteins were confirmed to be orthologous protein with the highest identities in each metabolic pathway using the BLASTP against NCBI protein database.
